# Design Perceptions for 3D Printed Accessories of Digital Devices and Consumer-Based Brand Equity

**DOI:** 10.3389/fpsyg.2019.02800

**Published:** 2019-12-10

**Authors:** Yuke Meng, Muhammad Waseem Bari

**Affiliations:** ^1^School of Art and Design, Beijing Institute of Technology, Beijing, China; ^2^Lyallpur Business School, Government College University, Faisalabad, Pakistan

**Keywords:** design perception, brand equity, experiential value, 3D printing, smartphone accessories, PLS-SEM

## Abstract

Drawn on means-end theory and relationalism, this study investigates the impact of 3D printed branded accessories’ design perception on consumer-based brand equity (CBBE). In addition, how experiential value mediates the relationship between product design perception and CBBE. Based on a random sampling approach, the present study collected 535 3D printed branded accessories users’ responses through social media (WeChat). The data were analyzed through partial least squares, structural equation modeling (PLS-SEM) approach, smart PLS-3. The results reveal that two dimensions of design perception (functional and kinesthetic) have a significant impact on CBBE. However, design perception (visual) has failed to impact significantly on CBBE. The experiential value significantly mediates the relationship between all dimensions (visual, functional, and kinesthetic) of design perception and CBBE. This study concludes that experiential value/user experience is an inevitable mediator between product design perception and CBBE. The implication and limitations of this study are discussed in the last section of this study.

## Introduction

In a contemporary business environment, every organization strongly believes in branding and brand equity. Organizations prioritize to develop and sustain brand equity by considering it a priceless asset for the organization (Raji et al., 2019). Consumer perspective, organizational perspective, and financial perspective three different approaches are in practice to measure the brand equity (Farjam and Hongyi, 2015). Consumer-based brand equity (CBBE) integrates the customer’s point of view to understand and measure brand equity (Baalbaki and Guzmán, 2016; Raji et al., 2019). In previous studies, scholars concluded incoherent outcomes from the relationship between CBBE and product design perception (Noble and Kumar, 2008, 2010; Mishra et al., 2015). For instance, Mishra et al. (2015) described that product design perception and CBBE have no significant direct relationship, however, consumer experimental value mediates the impact of product design perception and CBBE. Mishra conducted another study and observed that design perception, user experience, and CBBE have strong direct and indirect relationships (Mishra, 2016). Mishra conducted the first study on digital devices and the second one on different interactive electronic devices (Mishra et al., 2015; Mishra, 2016). These incoherent results raise several queries. For example, is there any significant relationship between product design perception and CBBE or not? Secondly, whether consumer experiential value is an inevitable mediator between design perception and CBBE?

Drawn on means-end theory, a buyer shapes a subjective understanding of product topographies at an abstract level with restricted information about explicit characteristics (Gutman, 1982; Mishra et al., 2015). Customer perception about design is the beginning stage of a dyadic connection between the item and the consumer (Mishra et al., 2015). It is very difficult for a designer and marketing staff alike to comprehend this perception to confirm product success in the market. Design is one of the key concerns before buying and regular usage of a product. Digital devices such as smartphones, digital cameras are usually expensive products and buyers are much concerned about design beauty, functionality, and visuality. Similarly, customers require such types of accessories like mobile casing, glass tampers, device holding which not only sustained but also enhance the beauty and protection of the device.

Based on design value model of Noble and Kumar (2010) and structure of web design of Garrett (2011) and Mishra et al. (2015) proposed five dimensioned model namely, visual design, functional design, kinesthetic design, interface design, and information design to measure the design perception of digital devices by a consumer. Mishra et al. (2015) and other scholars clarified that these five dimensions are only suitable for digital devices and web designing. However, for design perception measurement of general products, like 3D printed mobile phone accessories, only the first three dimensions (visual, functional, and kinesthetic) can be used (Sonderegger and Sauer, 2010; Mishra et al., 2015). The visual design properties comprise with sophistication, style, simplicity, color, material, and finish of the 3D printed device accessories, right size, soft on eyes, soft edges, eye-catching, and matching user’s personality. Functional design properties include newest topographies, good competitor, rough and tough; long-serving, easy to apply on the device, easy-going, multipurpose support, and reliable for the device. kinesthetic design properties include easy to relocate, adjustable in the pocket and device bags, smooth and supporting keypad, lightweight, simple, proper indications and locations of key parts of digital device.

The accessories of digital devices such as computers, mobile phones, video cameras, and video games are highly impacted by 3D printing technology. 3D printed accessories of mobile phones such as casing, holders, hands-free, and tempered screen glass have also been attracted the customers worldwide. Currently, in 2019, mobile phone accessories sales have more than 74 billion US dollars which are expected to cross 93 billion US dollars in 2023 (Holst, 2019). Several global brands of mobile phones such as Nokia, Samsung, and iPhone have also introduced branded 3D printed mobile phone accessories. Even, Nokia and iPhone have introduced 3D customized mobile phone accessories such as casings, phone holdings, etc. (Rindfleisch and O’Hern, 2015).

Based on consumption value theory (Sheth et al., 1991) and theory of consumption (Holbrook, 1999), the literature shows that user experience about the product design is more significant for CBBE than just a perception of consumer about a product design (Sheng and Teo, 2012; Mishra et al., 2014, 2015; Ding and Tseng, 2015; Mishra, 2016). This study interested to investigate the mediating role of consumer experiential value between product design perception and CBBE by considering the case of 3D printed branded accessories of branded smartphones. Keeping in view the importance of product design perception and its incoherent relationship with CBBE in literature. The present study is interested to evaluate the design perception of 3D printed branded accessories of digital devices (mobile phones) impact on CBBE. In addition, to determine the role of experiential value between product design perception and CBBE is the second concern of the present study. To sought these objectives, the present study has the following research questions. What is the impact of consumer design perception on CBBE and how experiential value mediates the relationship of consumer design perception and CBBE? For the empirical investigation, the users of 3D printed branded accessories of smartphones are employed in this study.

### Literature Review and Hypotheses Development

#### 3D Printed Branded Smartphone Accessories

3D printing/additive manufacturing is a progressive technology that has changed the two centuries-old approaches of designing and assembling in the perspectives of social, economic, environmental, and demographic implications (Campbell et al., 2011). 3D printing refers to a process of manufacturing in which different materials such as metal or plastic, are dropped in layers to yield a three dimensional (3D) entity (Schubert et al., 2014), for instance, cellular phone casings, keypads, holdings, eyeglasses, etc. 3D printed objects and their designs are more fascinating and economical at a micro-level as compare to the traditional manufacturing process (Schubert et al., 2014). 3D printing technology is contributing to different fields such as engineering, healthcare, education, architecture, and fashion (Jordan, 2019; Kim et al., 2019). 3D printing technology is equally capable to decrease not only financial cost (production, packing, transportation, and distribution) but also environmental cost (Campbell et al., 2011). Additive manufacturing technology provides customized designs and efficient manufacturing. 3D printed technology has transformed mass production into mass customization because each product produced through 3D technology is customized without any additional cost or a little additional cost (Campbell et al., 2011). Last two decades, several companies such as “LULZBOT” have been involved in 3D printed accessories of smartphones such as device protection cases, Bluetooth speakers, and battery packs, etc. (Saunders, 2017). In the future, 3D printing technology will grasp the major share of mobile phone accessories in the industry (Kietzmann et al., 2015).

#### Product Design Perception and Consumer Based Brand Equity

The first impression of a product for a consumer is its design and the strength of the first impression of the design affects the buying decision (Baek and Michael, 2017). Mishra et al. (2015) explained that after buying consumers also assess design value during consuming the product. Several cleared that product design is a complete summary of multiple activities in the production phase and a fundamental reason to buy and consume a product (Mishra et al., 2015; Baek and Michael, 2017). Product design is an influential strategic tool not only for brand development but also for its sustainability (Best, 2008; Brunner et al., 2008). Bloch (1995) highlighted that the design of a product provides worthwhile information to support users for the development of their initial impression toward a product (Baek and Michael, 2017).

There are several pieces of evidence in the literature that indicate the importance of product design and its impact on customers’ reactions and loyalty (Bloch, 1995; Mishra et al., 2015; Mishra, 2016; Baek and Michael, 2017). There are different approaches are recommended to measure product design perception. For instance, Bloch (1995) proposed a way to product design, suggesting a framework of customer responses based on behavioral and psychological elements. Afterward, a holistic way to design was developed as a blend of feel and look, functionality, and end consumer needs (Venkatesh and Meamber, 2006; Baek and Michael, 2017). Later, three dimensions of design namely aesthetics, functionality, and symbolism were proposed which have been extensively applied in the literature (Noble and Kumar, 2008; Homburg et al., 2015; Mishra et al., 2015; Baek and Michael, 2017).

Recently, Mishra et al. (2015) proposed and empirically investigated five dimensions based design perception model to measure the digital devices. Mishra derived three dimensions namely, visual esthetics, features and graphics, the kinesthetic value from the *design value* model of Noble and Kumar (2010), and two dimensions namely, interface and information design from the design perception framework “*Structure of Web Design”* developed by Garrett (2011). The key properties of these five dimensions as explained by Mishra et al. (2015) are as follows. The visual design includes elegance, simple and stylish, sober, attractive colors, proper size, round and soft edges, looks attractive in total, and suits the user’s personality. Functional design features include the latest and all good basic functions, better features from the competitors, durable and long life, multiple possible features, smooth in use, and dependent able with low failures. Kinesthetic design properties include adjustable in pocket and travel, reasonable keypad, frivolous, simple, main points properly indicated, easy to link with other devices. Interface design features include a customizable interface, responsive, easy-going and understandable, user-friendly, fulfill user requirements. Lastly, information design properties include useful information presented properly, clear and simple language, readable, understandable logos, and simple instructions. Mishra et al. (2015) clarified that last two dimensions of design perception are suitable only for digital devices. However, first three dimensions are suitable for all general products. The present study has focused on the design perception of 3D printed branded smartphone accessories. Thus, the focus of the present study is on the first three dimensions (visual, functional, and kinesthetic) of design perception model as independent variables.

A combination of brand liabilities and assets associated to a brand, its title, and logo that increase/decrease from the worth provided by a service/product to a company or company s’ customers or both refer to brand equity (Aaker, 1996; Sürücü et al., 2019). In fact, brand equity is a type of commercial value that arises from consumer perception about the brand name of a specific product/service, instead of product/service itself (Sürücü et al., 2019). Literature explained five dimensions of brand equity namely, perceived quality, brand association, brand loyalty, proprietary brand assets, and brand awareness (Aaker, 1991; Mishra et al., 2015; Sürücü et al., 2019). Keller (1993) considered the perspective of cognitive psychology and explained the differential impact of brand information on customer reaction to the marketing endeavors of the brand denoted as CBBE. Brand knowledge based on brand image and brand awareness plays a significant role in the development of CBBE (Keller, 1993; Sürücü et al., 2019). Lassar et al. (1995) defined that the increase in attractiveness and perceived value of the brand name refers to CBBE. Lassar et al. (1995) explained the five dimensions of the CBBE scale namely, value, social image, trustworthiness, performance, and commitment (Sürücü et al., 2019).

Several authors use brand equity and CBBE interchangeably and these scholars consider the value generated by marketing actions as perceived by customers (Mackay et al., 1998; Mohan and Sequeira, 2016). The operational perspective of CBBE has two approaches i.e., cognitive approach (consumer perceptions) and behavioral approach (consumer behavior) (Silverman et al., 1999). The key features of the cognitive approach include brand associations, brand awareness, and perceived quality, and behavioral approach includes price consciousness and brand loyalty (Myers, 2003; Mohan and Sequeira, 2016). Keller (2003) explained that the intensity of the brand lies in what consumers have realized, felt, seen, and caught wind of the brand because of their encounters after some time (Mohan and Sequeira, 2016).

The smartphone accessories such as casing, phone holders, hands-free, and tempered screen glass are considered an integral part of smartphones by the users (Rachel, 2016; Filieri and Lin, 2017). Several smartphone users have contrary arguments. The users consider that phone casings and screen protectors are important for expensive digital devices, however, these accessories hide the grace of the device and undermine the user personality (Paul and Yordan, 2018). By acknowledging the concerns of the users regarding smartphone accessories, several renown smartphone brands have started to offer 3D printed customized casings, screen protectors and other relevant accessories (Krassenstein, 2014). The design of 3D printed branded smartphone accessories has much attraction and demand in the market. 3D printed branded accessories are elegant, supportive and keep all functions of smartphones active, adjustable with the device, and no hassles to carry them. Several authors concluded that product design perception impacts the CBBE (Mishra et al., 2015; Mishra, 2016; Baek and Michael, 2017; Filieri and Lin, 2017; Jordan, 2019).

Drawn on the paradigm of relationalism and means-end theory, by considering the case of 3D printed branded smartphone accessories, the focus of the present study is to evaluate the impact of different product design perception on CBBE. Thus, the present study hypotheses that,

H1: Consumer design perception (Visual) has positive impact on CBBE.

H2: Consumer design perception (Functional) has positive impact on CBBE.

H3: Consumer design perception (kinesthetic) has positive impact on CBBE.

#### Experiential Value/User Experience

Experiential value is denoted as a client’s observation dependent on interactions including either direct utilization or separated gratitude of goods and services (Mathwick et al., 2001; Mishra et al., 2015). The framework of user experience/experimental value is measured with three dimensions namely, usability, social value, and pleasure in use (Holbrook, 1999; Mishra et al., 2014). The usability and pleasure in use refer to utilitarian value and hedonic value respectively. Nielsen (1993) explained that user experience is observed with two angles, first is the utilitarian value (effectiveness and efficiency in task completion) and second includes memorability, satisfaction, learnability, and easiness with a product (Mishra et al., 2015). Social value develops when consumer s’ self-utilization behavior performs as a source for molding the reactions of others (Mishra et al., 2014). Products are considered the well-recognized platform and cause social interpretation and impressions (McDonagh et al., 2002). Bandura (1986) argued that future consumption behavior highly depends on interactions and the environment where the individuals interact. Scholars explained that along with usability and sociability, a product also needs to be pleasurable (Mishra et al., 2014, 2015; Mishra, 2016). Pleasure in use refers to an end condition of experience where the appearance of positive emotions because of product use causes the customer to feel pleasure (Jordan, 1998). Generally, a product develops a relationship with the customer and leads to emotional value (Crilly et al., 2004; Mishra et al., 2015), and emotions and pleasure are used reciprocally (Russell and Lanius, 1984; Mishra et al., 2014).

The present study proposes experiential value based on its three dimensions (usability, social value, and pleasure in use) as a mediator between consumer design perception and CBBE. Several studies have conducted in different contexts where experiential value is used as dependent on design perception and a mediator between design perception and CBBE. However, the results of these studies are incoherent. For instance, Mishra et al. (2014) investigated the association between consumption values/user experience and brand equity by getting the response from smartphone users, and results confirmed the significant association. Mishra et al. (2015) evaluated the mediating role of user experience between five dimensions of product design perception and CBBE by getting the response from digital devices users. The results depicted that user experience value significantly mediates the relationship between design perception and CBBE. The same results have repeated in a study on smartphones conducted by Mishra (2016). Ding and Tseng (2015) conducted a study on four big service brands and concluded that hedonic emotions mediate the relationship between brand experience and customer brand loyalty. Sheng and Teo (2012) also conducted on smartphone users, the results proved that user experience plays an important role of a bridge between product attributes and brand equity. Foroudi et al. (2016) also conducted a study on 606 consumers of international retail brands. The results confirmed a significant association between consumer experience and firm loyalty.

Drawn on consumption value theory (Sheth et al., 1991) and theory of consumption (Holbrook, 1999), this study interested to investigate the mediating role of consumer experiential value between product design perception and CBBE by considering the case of 3D printed branded accessories of branded smartphones. Hence, it is hypothesized that,

H4: Experiential value mediates the association between Consumer design perception (Visual) and CBBE.

H5: Experiential value mediates the association between Consumer design perception (functional) and CBBE.

H6: Experiential value mediates the association between Consumer design perception (kinesthetic) and CBBE.

**FIGURE 1:**
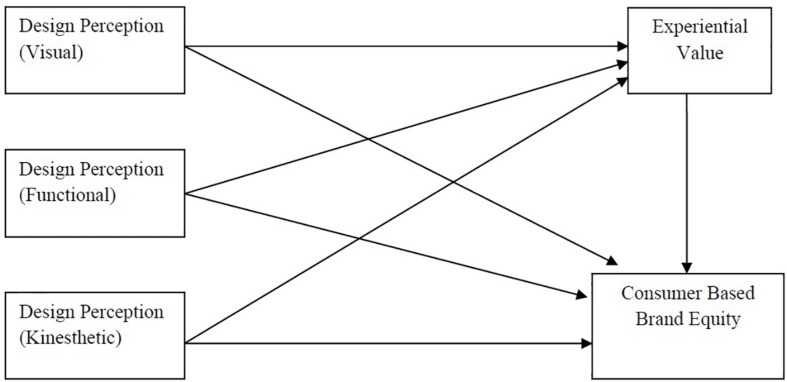
Study framework.

Figure 1 explain the relationship between variables and study framework.

## Materials and Methods

### Sample and Data Collection Procedure

The present study applied an online survey approach using social media “WeChat.” WeChat is the most popular social media platform in China and almost every smartphone holder in China is active on WeChat and they are also aware of the 3D printed smartphone accessories. Hence, WeChat is a suitable platform for getting a response for the present study model. The authors created a new closed group at WeChat with the study title. The authors requested the university graduate students and members of other WeChat groups for joining our specific study group. While requesting to others, authors cleared the objectives of joining this study group at WeChat. One month continuously request to WeChat users at the university campus and at different WeChat groups, a total of 708 individuals randomly joined the WeChat group. After 708 members, the group was banned and closed for new entries.

After closing the membership campaign, the authors allocated the specified digital code to each member instead of their contact names (profile names) on the WeChat group. After this activity, all respondents’ recognition at the WeChat group was just a specified code only. The codification was also a signal toward group members that you will not be recognized with your response. The codification was also helpful to reduce bias in respondents. At a specified date and preintimation to the group members, the authors uploaded the questionnaire in the group in dual languages (English and Chinese) with a cover letter. In the cover letter, the respondents were assured that their response will be used only for this study purpose and all sorts of your recognition will be kept secret. In addition, it was also requested to the group members for sending their responses file as a private message to the authors at WeChat. The cover letter also assured the participants that there is no right or wrong answer and requested that the participants should respond to the questions just naturally. This research paper also took on board the issue of non-response bias possibility (Dillman, 1991). The authors addressed this issue through comparison with early and late respondents. Post-comparison of the results of early respondents and late respondents (who sent a response after reminder) confirmed that there is no significant difference in responses. These procedural activities supported to reduce social desirability/acquiescence biases (Spector, 2006; Shaheen et al., 2019).

The present study used “Harman’s Single-Factor Test” to examine the common method variance (CMV). A Harman one-factor analysis is a *post hoc* technique that is performed post-data collection to examine whether a single factor is liable for smartphones in the data set (Tehseen et al., 2017). The present study used SPSS 21 to perform the “Harman’s Single-Factor Test.” The generated “Principal Component Analysis” output showed 34 distinct factors accounting for 65% of the total variance (Supplementary Appendix 1). The 1st unrotated factor taken just 47% of the variance in data set. Therefore, the two criteria/assumptions did not fulfill. First, no single factor developed. Second, the first factor did not cover most of the variance (Tehseen et al., 2017). Thus, the results convey that CMV is not a problem in this study.

The present study used a time lag data collection approach; thus, the data were collected in three ways. Wave 1, the questions were asked to 708 individuals about three dimensions of design perception and demographics of respondents. Out of those 708, 644 respondents provided the complete answers. The non-respondent (64) codes were deactivated in the group. Wave 2, after 30 days gap, the questions about CBBE were asked to active 644 individuals. However, from these 644, 590 respondents provided the answers. The non-respondent (54) codes were deactivated in the group. Wave 3, with a further 30 days gap, the questions about user experiential value were asked to 590 members. Finally, 535 respondents provided answers to the completed questionnaire. The non-respondent (55) codes were deactivated in the group. Thus, the attrition rates in Wave 1, Wave 2, and Wave 3 were 9, 8.39, and 9.32% respectively, which are acceptable in an online data collection method (Dillman, 2011). This study was also permitted by the ethics committee of the Beijing Institute of Technology, Beijing, China. Moreover, the data were collected on the free consent and readiness of the participants without any social or professional influence.

### Measurement of Variables

The empirically well-established scales are used to measure the variables of this study. The items of the scale were reasonably changed by considering the 3D printed branded smartphone accessories and Chinese context. This scale was first used by Mishra et al. (2015) to measure the different dimensions of consumer design perception. Mishra et al. (2015) used this scale to measure consumer design perception for digital devices such as smartphones. The present study has measured the consumer design perception of 3D printed accessories for smartphones. Therefore, keeping the theme of consumer design perception about 3D printed accessories, the changes are made in the scale developed by Mishra et al. (2015). For instance, “*my devices styling looks elegant”* (original). “*The styling of my phone holder looks elegant”* (revised). The items of all variables are anchored on a 5-points Likert scale (strongly disagree = 1 to strongly agree = 5). A complete questionnaire is given as Supplementary Appendix 3.

#### Consumer Design Perception (Visual)

Consumer design perception (visual) is measured with 5 items scale developed by Mishra et al. (2015). For instance, “*The styling of 3D printed accessories (Casing and holder) of my phone looks elegant.”* Cronbach’s Alpha value is 0.849.

#### Consumer Design Perception (Functional)

Consumer design perception (functional) is measured with 7 items scale developed by Mishra et al. (2015). For example, *“My phone’ 3D printed casing offers the right number of basic features that I need.”* Cronbach’s Alpha value is 0.821. After the pilot study, one item was deleted from the final model due to lower outer loading values.

#### Consumer Design Perception (kinesthetic)

Consumer design perception (kinesthetic) is measured with 3 items scale developed by Mishra et al. (2015). For example, *“The size of 3D printed accessories (Casing and holder) of my phone makes it easy to carry and move around.”* Cronbach’s Alpha value is 0.833.

#### Experiential Value

Experiential Value is measured with 19 items scale developed by Mishra et al. (2015). For instance, “*I use 3D printed branded accessories for my phone frequently.”* Infect, experiential value scale based three dimensions (usability, pleasure in use, and social value) and three different scales originally developed by Brooke (1996) and Sweeney and Soutar (2001). Cronbach’s Alpha value is 0.920. After the pilot study, 3 items were deleted from the final model due to lower outer loading values.

#### Consumer-Based Brand Equity

Consumer-based brand equity is evaluated with 4 items scale developed by Yoo and Donthu (2001) and Mishra et al. (2015). For instance, *“It makes sense to choose the 3D printed phone accessory (casing/holder) of this brand instead of any other, even if they are the same.”* Cronbach’s Alpha value is 0.885.

### Statistical Model

The present study applied a variance-based partial least squares structural equation modeling approach (PLS-SEM) instead of co-variance-based approach such as AMOS, LISREL, because PLS-SEM supports both confirmatory and exploratory research (Chwelos et al., 2001; Sheng and Teo, 2012; Hair et al., 2016). PLS-SEM is a popular approach used to analyses the data on contemporary issues of marketing (Sheng and Teo, 2012; Junni et al., 2015). PLS-SEM is quite suitable for complex and multi-order models (Hair et al., 2016, 2019). PLS-SEM with small data size also performs well (Bari et al., 2019). PLS-SEM considers all path coefficients and item loadings while analyzing the data, which decreases parameter estimate biases (Fornell and Larcker, 1981; Sheng and Teo, 2012; Hair et al., 2016). Smart Pls-3 software is used for the application of PLS-SEM.

## Results and Analyses

### Respondents Profile

Table 1 explains the 535 respondents’ profile. In this study, out of 535 respondents, 61% male and 39% were female. Most of the respondents (64%) in this study were between the age of 20–35 years. Almost, half of the participants’ education level was undergraduate. This study was conducted in China, therefore, 94% of the total respondents were Chinese and remaining were the students of different nationalities who were studying in China.

**TABLE 1:** Respondents profile *N* = 535.

**Variables**	**Category**	**Frequency**	**Percentage**
Gender	Male	326	61%
	Female	209	39%
Age	20–35 years	342	64%
	36–50 years	145	27%
	51 and above	48	09%
Education	Under Graduate	300	56%
	Masters	198	37%
	Ph.D.	37	07%
Nationality	Chinese	503	94%
	Miscellaneous	32	06%

### Model Measurement

The reliability of the constructs was calculated using Cronbach’s alpha. An appropriate level of model reliability requires greater than 0.7 value of Cronbach’s alpha (Hair et al., 2016). Table 2 shows that all variables’ values of Cronbach’s alpha are higher than 0.7. As proposed by Fornell and Larcker (1981) Convergent validity of the model is assessed through composite reliability (CR), average variance extract (AVE), and items reliability of each variable (outer loadings). As recommended by the experts, CR and AVE values of each construct should be higher than 0.6 and 0.5 respectively (Fornell and Larcker, 1981; Hair et al., 2016). Table 2 confirm that all values of CR and AVE are meeting the criteria. The factor loadings of all items at the individual level should be higher than 0.7. However, if few items have values slightly less than 0.7 (Sarstedt et al., 2014), they can be taken into account if other criteria such as CR, AVE, and Cronbach’s alpha are fulfilled (Hair et al., 2016). Table 2 depicts that all outer loadings are higher than 0.7.

**TABLE 2:** Model measurement.

**Constructs**	**Items**	**Mean**	***SD***	**Kurtosis**	**Skewness**	**Outer loading**	**α**	**rho_A**	**CR**	**AVE**
Design perception (visual)	DPV-1	2.303	1.172	–0.387	0.623	0.771	0.849	0.864	0.891	0.620
	DPV-2	2.426	1.164	–0.458	0.525	0.788				
	DPV-3	2.099	1.102	0.199	0.887	0.827				
	DPV-4	2.204	1.118	0.005	0.791	0.833				
	DPV-5	1.931	0.988	0.256	0.873	0.713				
Design perception (functional)	DPF-1	2.136	1.103	–0.111	0.767	0.661	0.821	0.832	0.869	0.527
	DPF-2	2.579	1.126	–0.586	0.305	0.680				
	DPF-3	2.422	1.101	–0.330	0.522	0.721				
	DPF-4	2.411	1.071	–0.257	0.470	0.736				
	DPF-5	2.396	1.178	–0.509	0.542	0.765				
	DPF-6	2.204	1.138	–0.237	0.716	0.784				
Design perception (kinesthetic)	DPK-1	1.759	1.075	1.780	1.534	0.893	0.833	0.835	0.881	0.750
	DPK-2	1.873	1.050	0.792	1.190	0.852				
	DPK-3	2.022	1.132	–0.031	0.925	0.852				
Experiential value	EV-1	1.996	1.070	0.041	0.881	0.771	0.950	0.949	0.947	0.573
	EV-2	2.026	0.991	0.173	0.802	0.789				
	EV-3	2.036	1.016	0.014	0.765	0.789				
	EV-4	2.187	1.026	–0.109	0.619	0.750				
	EV-5	2.357	1.072	–0.080	0.585	0.655				
	EV-6	2.308	1.106	–0.421	0.524	0.670				
	EV-7	2.189	1.111	–0.254	0.697	0.713				
	EV-8	2.101	1.063	–0.004	0.781	0.804				
	EV-9	2.084	1.062	–0.079	0.752	0.799				
	EV-10	2.196	0.990	–0.155	0.526	0.788				
	EV-11	2.133	1.004	0.063	0.653	0.736				
	EV-12	2.019	1.034	–0.108	0.756	0.743				
	EV-13	2.088	1.038	–0.361	0.617	0.742				
	EV-14	2.200	1.062	–0.128	0.645	0.728				
	EV-15	2.073	1.005	–0.089	0.685	0.797				
	EV-16	1.929	1.021	0.077	0.894	0.819				
Consumer based brand equity	CBBE-1	2.127	0.993	–0.371	0.536	0.867	0.885	0.885	0.920	0.743
	CBBE-2	2.125	1.018	0.000	0.695	0.855				
	CBBE-3	2.058	1.076	0.121	0.860	0.863				
	CBBE-4	2.103	1.090	0.153	0.829	0.862				

**DPV, design perception visual; DPF, design perception functional; DPK, design perception kinesthetic; EV, experiential value; CBBE, consumer based brand equity; CR, composite reliability; AVE, average variance extracted;*α, *Cronbach’s Alpha; SD, standard deviation.**

The present study assessed the discriminant validity through two approaches namely, Fornell-Larcker Criterion and Heterotrait-Monotrait Ratio (HTMT). As per Fornell and Larcker (1981) criterion, this study tested the discriminant validity of the variables by investigating whether the square root of the AVE of each variable is higher than the highest correlation among the latent variable concerning the principal variable (as bold figures are shown in Table 3), signifying discriminant validity has been establishing. As per the second criterion, the HTMT ratio should be less than 0.90 for the establishment of model discriminant validity (Hair et al., 2016). Table 3 confirm that all HTMT ratios are less than 0.90.

**TABLE 3:** Discriminant validity.

**Fornell-Larcker criterion**						**Heterotrait-Monotrait ratio (HTMT)**				
**Constructs**	**CBBE**	**DPF**	**DPK**	**DPV**	**EV**	**Constructs**	**CBBE**	**DPF**	**DPK**	**DPV**
CBBE	0.862					CBBE				
DPF	0.685	0.726				DPF	0.786			
DPK	0.616	0.538	0.866			DPK	0.716	0.634		
DPV	0.680	0.711	0.580	0.787		DPV	0.745	0.830	0.669	
EV	0.780	0.680	0.626	0.723	0.757	EV	0.872	0.810	0.699	0.762

**DPV, design perception visual; DPF, design perception functional; DPK, design perception kinesthetic; EV, experiential value; CBBE, consumer based brand equity.**

Table 4 explains the predictive capabilities of the model. All inner VIF values are significantly less than 5 (Hair et al., 2014) which confirms the robustness of the model. The *f*^2^ effect sizes of the constructs mostly at medium and higher levels which is also a positive sign for a strong model (Gefen et al., 2000; Hair et al., 2016). Greater than 0.5 *R*^2^-value indicates a substantial model, in the present study, *R*^2^-values of EV and CBBE are 0.653 and 0.729 respectively. *Q*^2^**** *(Cross-Validated Redundancy)* of both latent variables is considerably higher than 0 (zero) which is another evidence of the significance of the present study model.

**TABLE 4:** Model’s predictive capabilities.

**Measures**	**Constructs**	**DPF**	**DPK**	**DPV**	**EV**
VIF	EV	2.126	1.582	2.277	–
(inner values)	CBBE	2.534	1.748	2.565	2.885
*f*^2^	EV	o.192	0.105	0.127	–
(effect size)	CBBE	0.015	0.030	0.010	0.511
*R*^2^	EV	0.653			
(variance explained)	CBBE	0.729			
*Q*^2^	EV	0.347			
(cross-validated redundancy)	CBBE	0.509			

**DPV, design perception visual; DPF, design perception functional; DPK, design perception kinesthetic; EV, experiential value; CBBE, consumer based brand equity.**

### Direct Effects

This study examined the direct effects of three dimensions (visual, functional, kinesthetic) of product design perception on CBBE. Table 5, the results confirm that design perception (visual) (β = 0.082, *p* > 0.067) has no significant effect on CBBE, thus, H1 is not accepted. Contrary, other two dimensions of product design perception, functional (β = 0.102, *p* < 0.008) and kinesthetic (β = 0.119, *p* < 0.001) have significant association with CBBE. Therefore, H2 and H3 are accepted.

**TABLE 5:** significance of structural paths (direct effects).

**Structural paths**	**β-value/*(T* value)**	**Confidence intervals (bias corrected)**	***P*-value (0.05 level of significance)**	**Outcomes**
DPV → CBBE	0.082, (1.833)	(0.000–0.176)	0.067	H1 = Not Supported Due to incomplete information
DPF → CBBE	0.102, (2.665)	(0.022–0.172)	0.008	H2 = Supported
DPK → CBBE	0.119, (3.442)	(0.053–0.187)	0.001	H3 = Supported

**DPV, design perception visual; DPF, design perception functional; DPK, design perception kinesthetic; CBBE, consumer based brand equity., All values are significant at 0.050% (2-tailed).**

### Direct Effects/Mediation

**FIGURE 2:**
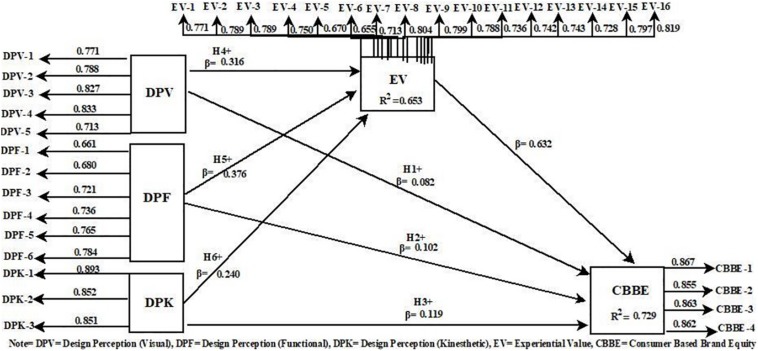
Developed model.

Table 6 explains the mediation analyses of the present study model. For the calculation of the mediation effect in the present study, VAF (variance accounted for) approach is applied (Hair et al., 2013; Ali and Park, 2016; Bari et al., 2016). The experimental value as a mediator significantly partially mediates the relationship between three dimensions (visual, functional, and kinesthetic) of product design perception and CBBE. It is also noticeable here that the direct relationship between product design perception (Visual) and CBBE was not significant (see Table 5). However, after adding the experiential value as a mediator, product design perception (visual) has a strong indirect effect on CBBE than the other two dimensions of design perception. Therefore, H4, H5, and H6 are accepted. Figure 2 also explains the key results after an empirical investigation of the present study model.

**TABLE 6:** Significance of structural paths (indirect effects).

**Structural paths**	**Direct effect/*(T* value)**	**Indirect effect (*T-*value)**	**Total effect**	**Confidence intervals of total effect**	**VAF %**	**Interpretation**
DPV → EV → CBBE	0.082, (1.833)	0.200 (6.707)	0.282 (5.829)	(0.179–0.383)	70.92	H4 = Supported partial mediation
DPF → EV → CBBE	0.102, (2.665)	0.238 (7.957)	0.339 (7.718)	(0.256–0.426)	70.20	H5 = Supported partial mediation
DPK → EV → CBBE	0.119, (3.442)	0.152 (5.939)	0.270 (6.491)	(0.193–0.346)	56.29	H6 = Supported partial mediation

**DPV, design perception visual; DPF, design perception functional; DPK, design perception kinesthetic; EV, experiential value; CBBE, consumer based brand equity. All values are significant at 0.05% (2-tailed), VAF, variance accounted for, The VAF > 80% indicates full mediation, 20% ≤ VAF ≥ 80% shows partial mediation while VAF < 20% as no mediation (Bari et al., 2016).**

## Discussion

The objective of the present study was to investigate the impact of 3D printed accessories’ (of branded mobile phones) design on CBBE. In addition, how experiential value (user experience) enhances the impact of product design perception on CBBE. This study investigated the impact of three dimensions (visual, functional, and kinesthetic) of product design perception on CBBE separately. At the WeChat group, the data collected from 535 users of 3D printed accessories of branded phones revealed very interesting results. The results confirmed the propositions of the present study that functional and kinesthetic aspects of the 3D printed accessories smartphones increase the CBBE. Several previous studies’ results are also in line with these results (Sheng and Teo, 2012; Mishra et al., 2014; Rindfleisch and O’Hern, 2015; Mishra, 2016). Contrary, 3D printed smartphone accessories’ design perception (visual) does not significantly enhance CBBE. In the case of 3D printed branded smartphone accessories, the insignificant relationship between design perception (visual) and CBBE opened a new debate. This debate raises two questions. First, whether the buyers are more concerned about the functional supportive and easy-going accessories than the visuality of the accessories? Second, do users prefer the role of protection and functioning well of accessories over visuality of the 3D printed phone accessories?

The literature has a mixed response to the above-mentioned questions. For instance, several customers perceived that highly expensive branded smartphones need the utmost protection from falling, and supporting accessories should be configured properly with a phone (Rindfleisch and O’Hern, 2015; Babu, 2017). This response of customers indicates the importance of accessories’ design function and kinesthetic aspects as compare to design visuality. Contrary, some customers perceive branded smartphones such as the iPhone, Samsung, Xiaomi, and Nokia as a symbol of social status (Jordan, 1998; Krassenstein, 2014; Babu, 2017; Jordan, 2019). The visuality of the accessories like casing, hand-free headphones, phone holders, phone covers may affect the grace of the phone and social status of the phone owner. Thus, it is important that an accessory’ design (visual, function, and kinesthetic) of an expensive branded smartphone should be at par. Supplementary Appendix 2 depicts the pictures of 3D printed accessories of a smartphone.

The mediation results reveal that experiential value is an important aspect to determine the CBBE through product design. The analyses (Table 6) shows that experiential value significantly mediates the relationships between dimensions of product design (Visual 71%, functional 70%, and kinesthetic 59%) and CBBE. These results are also in line with (Mishra et al., 2015; Mishra, 2016). The post-user experience, the visuality of the product design becomes the most important aspect. In other words, product design perception (visual) and CBBE was not directly significantly associated. However, post-experience, consumers realize the importance of design visuality. The results also support the above-raised question that the users of the expensive branded phones are concerned about their social status. Up to perception, it is possible that product design (visual) is not an important aspect for the consumer. However, when a consumer gets experience and feedback from the community, the visuality of the product becomes very important. The results of the present study also acknowledge the strength of the theory of consumption value (Sheth et al., 1991; Holbrook, 1999; Mishra et al., 2015).

### Study Implications

#### Theoretical Implications

The present study extends the application of means-end theory (Gutman, 1982) and the theory of consumption value (Holbrook, 1999) by investigating the case of 3D printed branded accessories of smartphones. Based on relationalism, this study explains that how the design of 3D printed branded accessories is important to enhance the CBBE. Drawn on relationalism, the present paper linked the importance of phone accessories design with branded phones to enhance the CBBE. The present developed an innovative model to determine CBBE by integrating the two different theoretical perspectives, i.e., brand equity theory (Keller, 2003; Mishra et al., 2014) and theory of consumption value (Holbrook, 1999) with brand experience theory (Brakus et al., 2009). This study also helps in reducing the incoherency in the investigations of Mishra (Mishra et al., 2014, 2015; Mishra, 2016). The results of this study partially support the investigation of Mishra et al. (2015), the direct relationship between two dimensions of design perception and CBBE is significant and one is insignificant. However, experiential value/user experience significantly mediates the impact of all dimensions (visual, functional, and kinesthetic) of design perception on CBBE. Hence, it is strongly established that user experience is a fundamental mediator between consumer design perception and brand equity (Sheng and Teo, 2012; Mishra et al., 2015). By using the latest dimensions and items of design perception proposed by Mishra et al. (2015) for a general product, this study provided a microscopic view about the relationship between design perception and user experience, and CBBE.

#### Managerial Implications

A huge sale volume (74 billion US dollars) (Holst, 2019) of smartphone accessories has been confirmed that phone manufacturing companies cannot deny the importance of phone accessories. Therefore, big brands of smartphones are focusing on 3D printed branded accessories for smartphones (Krassenstein, 2014; Rindfleisch and O’Hern, 2015). Drawn on the outcomes of the present study, the following are the implications for practitioners and manufacturers. The first and clear point is that the success of product design highly depends on user experience. Therefore, the usage pleasure and social status of the customers should be a key focus of the 3D printed branded accessories manufacturers. Second, based on the responses of constructs items, the accessories should be simple to use, adjustable with the device, reliable, and completely supportive. Third, considering the social value perspective, a 3D branded accessory should be contemporary with great aesthetics, matching user and device personality, and attractive. Fourth, marketers also need to understand that only useable products cannot develop CBBE, it will be a risky and blind shot. Thus, it is necessary for professionals to also focus on user pleasure and social status while developing accessories. Fifth, all users of smartphones and their accessories are not experts and well educated, therefore, considering the aspect of usability, the designers should design the accessories simple, easy-going, and user-friendly.

#### Limitations and Future Directions

Like other studies, the present study also has some limitations, and future research directions as well. As per the best knowledge of the authors, this is the first study that investigated the 3D printed branded accessories of smartphones’ design impact on CBBE. 3D printed branded accessories are not very common in use and available in markets. Therefore, biases in data may be possible. This study has focused only on 3D printed branded accessories of smartphones. The model of the present study can be extended to the non-digital 3D printed products of other sectors such as 3D printed fashion and design products, 3D toys, etc. The present study collected the data at time lag bases. The total data collection period is around 3 months. In such a short period, it is difficult to judge consumer brand loyalty. Therefore, the present study model should apply on longitudinal data to confirm the following purchase intension/actual purchase by the consumer. The survey method is used for data collection of the present study which may produce biased outcomes because of common method variance. This deficiency may overcome through other data collection approaches, i.e., observation method (to confirm the repurchase behavior of consumer), laboratory test, and netnography (to investigate that how buyers are linked with the 3D printed branded accessories online at various social groups and forums to confirm the links envisioned in this study.

## Data Availability Statement

The datasets generated for this study are available on request to the corresponding author.

## Ethics Statement

The studies involving human participants were reviewed and approved by the School of Management and Economics, Beijing Institute of Technology. Written informed consent for participation was not required for this study in accordance with the national legislation and the institutional requirements.

## Author Contributions

YM developed the idea and research design, and drafted the manuscript. MB helped in data collection and performed the analysis. Both authors jointly revised and edited the final version of the manuscript several times.

## Conflict of Interest

The authors declare that the research was conducted in the absence of any commercial or financial relationships that could be construed as a potential conflict of interest.
