# Supplementary material for: Design Perceptions for 3D Printed Accessories of Digital Devices and Consumer-Based Brand Equity
Source: Front Psychol. 2019 Dec 10;10:2800. doi: 10.3389/fpsyg.2019.02800 (PMC6914854; doi:10.3389/fpsyg.2019.02800)
Supplement: Supplementary file 1 [file Data_Sheet_1.docx]

**Appendix 1**

**Harman’s single-Factor Test**

| **Extraction Method: Principal Component Analysis** | | | | | | |
| --- | --- | --- | --- | --- | --- | --- |
| **Component** | **Initial Eigenvalues** | | | **Extraction Sums of Squared Loadings** | | |
|  | **Total** | **% of Variance** | **Cumulative %** | **Total** | **% of Variance** | **Cumulative %** |
| 1 | 16.261 | 47.826 | 47.826 | 16.261 | 47.826 | 47.826 |
| 2 | 2.147 | 6.315 | 54.141 | 2.147 | 6.315 | 54.141 |
| 3 | 1.523 | 4.480 | 58.621 | 1.523 | 4.480 | 58.621 |
| 4 | 1.241 | 3.651 | 62.272 | 1.241 | 3.651 | 62.272 |
| 5 | 1.132 | 3.330 | 65.601 | 1.132 | 3.330 | 65.601 |
| 6 | .844 | 2.481 | 68.083 |  |  |  |
| 7 | .798 | 2.347 | 70.430 |  |  |  |
| 8 | .794 | 2.335 | 72.765 |  |  |  |
| 9 | .642 | 1.889 | 74.654 |  |  |  |
| 10 | .616 | 1.812 | 76.466 |  |  |  |
| 11 | .590 | 1.735 | 78.201 |  |  |  |
| 12 | .523 | 1.537 | 79.738 |  |  |  |
| 13 | .503 | 1.481 | 81.219 |  |  |  |
| 14 | .468 | 1.378 | 82.597 |  |  |  |
| 15 | .452 | 1.330 | 83.927 |  |  |  |
| 16 | .421 | 1.238 | 85.164 |  |  |  |
| 17 | .409 | 1.202 | 86.366 |  |  |  |
| 18 | .394 | 1.158 | 87.524 |  |  |  |
| 19 | .380 | 1.117 | 88.642 |  |  |  |
| 20 | .351 | 1.034 | 89.675 |  |  |  |
| 21 | .335 | .985 | 90.660 |  |  |  |
| 22 | .321 | .943 | 91.604 |  |  |  |
| 23 | .314 | .923 | 92.527 |  |  |  |
| 24 | .305 | .896 | 93.423 |  |  |  |
| 25 | .283 | .832 | 94.255 |  |  |  |
| 26 | .277 | .816 | 95.071 |  |  |  |
| 27 | .261 | .769 | 95.840 |  |  |  |
| 28 | .244 | .718 | 96.558 |  |  |  |
| 29 | .225 | .662 | 97.221 |  |  |  |
| 30 | .215 | .634 | 97.854 |  |  |  |
| 31 | .215 | .632 | 98.486 |  |  |  |
| 32 | .186 | .548 | 99.034 |  |  |  |
| 33 | .165 | .485 | 99.519 |  |  |  |
| 34 | .164 | .481 | 100.000 |  |  |  |

**Appendix 2**

**3D Printed Accessories of Smartphone**


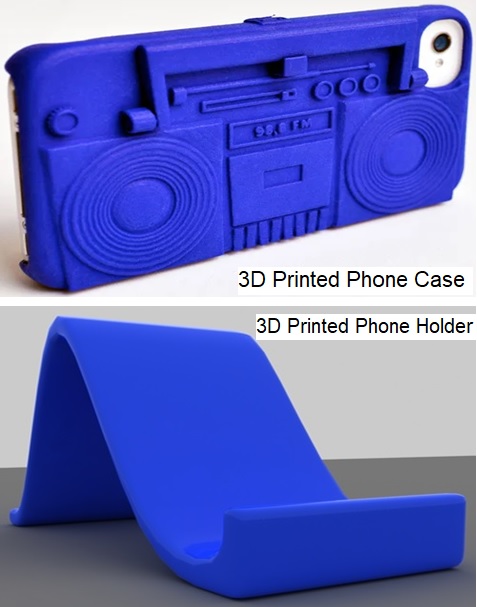


**Appendix 3**

**Design Perceptions for 3D Printed Accessories of Digital Devices and Consumer-Based Brand Equity**

1. **Consumer Design Perception**

**1.1- Visual** (Mishra, Dash, Malhotra, & Cyr, 2015)

Strongly Agree: 1 Agree: 2 Neutral: 3 Disagree: 4 Strongly Disagree: 5

| S. # | Items | Strongly Agree | Agree | Neutral | Disagree | Strongly Disagree |
| --- | --- | --- | --- | --- | --- | --- |
| 1 | The styling of 3D printed accessories (Casing and holder) of my phone looks elegant. | 1 | 2 | 3 | 4 | 5 |
| 2 | The appearance of 3D printed accessories (Casing and holder) of my phone is eye-catching. | 1 | 2 | 3 | 4 | 5 |
| 3 | Various elements of 3D printed accessories (Casing and holder) of my phone go well together. | 1 | 2 | 3 | 4 | 5 |
| 4 | My 3D printed accessories (Casing and holder) of my phone exhibit proper contrast through the right color combinations. | 1 | 2 | 3 | 4 | 5 |
| 5 | The personality of 3D printed accessories (Casing and holder) of my phone matches mine. | 1 | 2 | 3 | 4 | 5 |

**1.2- Kinesthetic (Reflective)** (Mishra, Dash, Malhotra, & Cyr, 2015)

Strongly Agree: 1 Agree: 2 Neutral: 3 Disagree: 4 Strongly Disagree: 5

| Sr # | Items | Strongly Agree | Agree | Neutral | Disagree | Strongly Disagree |
| --- | --- | --- | --- | --- | --- | --- |
| 1 | The shape and size of 3D printed accessories (Casing and holder) of my phone make it easy to use | 1 | 2 | 3 | 4 | 5 |
| 2 | The size of 3D printed accessories (Casing and holder) of my phone makes it easy to carry and move around. | 1 | 2 | 3 | 4 | 5 |
| 3 | The 3D printed accessories (Casing and holder) of my phone facilitate easy handling without physical or mental fatigue | 1 | 2 | 3 | 4 | 5 |

**1.3- Functional** (Mishra, Dash, Malhotra, & Cyr, 2015)

Strongly Agree: 1 Agree: 2 Neutral: 3 Disagree: 4 Strongly Disagree: 5

| Sr # | Items | Strongly Agree | Agree | Neutral | Disagree | Strongly Disagree |
| --- | --- | --- | --- | --- | --- | --- |
| 1 | The 3D printed accessories (Casing and holder) of my phone offer a lot of latest features. | 1 | 2 | 3 | 4 | 5 |
| 2 | My phone’ 3D printed casing offers the right number of basic features that I need. | 1 | 2 | 3 | 4 | 5 |
| 3 | My phone’ 3D printed holder is loaded with more features compared to its competitors. | 1 | 2 | 3 | 4 | 5 |
| 4 | My phone’ 3D printed casing offers seamless performance without glitches. | 1 | 2 | 3 | 4 | 5 |
| 5 | Technical specifications of my phone casing allow for running two or more applications. | 1 | 2 | 3 | 4 | 5 |
| 6 | I can always depend on 3D printed accessories (Casing and holder) performance of my phone. | 1 | 2 | 3 | 4 | 5 |
| 7 | *My phone’ casing is tough and can take a lot of abuse* | 1 | 2 | 3 | 4 | 5 |

*Note: One Italic item (7) have lower outer loading values and are deleted from the final model.*

**2- Experiential Value (**Mishra et al., 2015; Brooke, 1996; Sweeney & Soutar, 2001)

Strongly Agree: 1 Agree: 2 Neutral: 3 Disagree: 4 Strongly Disagree: 5

| Sr # | Items | Strongly Agree | Agree | Neutral | Disagree | Strongly Disagree |
| --- | --- | --- | --- | --- | --- | --- |
| 1 | I use 3D printed phone accessories frequently | 1 | 2 | 3 | 4 | 5 |
| 2 | I find the 3D printed phone accessories *(casing/holder)* simple to use | 1 | 2 | 3 | 4 | 5 |
| 3 | I can accomplish the task on phone more quickly with the help of 3D printed phone accessories | 1 | 2 | 3 | 4 | 5 |
| 4 | I frequently need the help of an expert to be able to use 3D printed phone accessories* | 5 | 4 | 3 | 2 | 1 |
| 5 | I find the operating options of 3D printed phone accessories well integrated. | 1 | 2 | 3 | 4 | 5 |
| 6 | I feel there is too much inconsistency in the 3D printed accessories’ functions* | 5 | 4 | 3 | 2 | 1 |
| 7 | *I find 3D printed accessories of phone easy to learn* | 1 | 2 | 3 | 4 | 5 |
| 8 | I find 3D printed accessories of phone awkward to use* | 5 | 4 | 3 | 2 | 1 |
| 9 | *I feel very confident using 3D printed accessories (casing/holder) of the phone.* | 1 | 2 | 3 | 4 | 5 |
| 10 | I needed to learn a lot of things before I started using 3D printed accessories (casing/holder) of phone* | 5 | 4 | 3 | 2 | 1 |
| 11 | The 3D printed accessories (casing/holder) of the phone makes me feel acceptable in a group | 1 | 2 | 3 | 4 | 5 |
| 12 | The 3D printed accessories (casing/ holder) of phone improves the way I am perceived | 1 | 2 | 3 | 4 | 5 |
| 13 | The 3D printed accessories (casing/holder) of my phone makes a good impression on other people | 1 | 2 | 3 | 4 | 5 |
| 14 | The 3D printed accessories (casing/ holder) of my phone enhances my social status | 1 | 2 | 3 | 4 | 5 |
| 15 | The 3D printed accessories (casing/ holder) of my phone is the one I really enjoy while using | 1 | 2 | 3 | 4 | 5 |
| 16 | *The 3D printed accessories (casing/ holder) of my phone always makes me want to use it* | 1 | 2 | 3 | 4 | 5 |
| 17 | I feel very relaxed while using 3D printed accessories (casing/ holder). | 1 | 2 | 3 | 4 | 5 |
| 18 | The 3D printed accessories (casing/ holder) of my phone makes me feel very good | 1 | 2 | 3 | 4 | 5 |
| 19 | The 3D printed accessories (casing/ holder) of the phone gives me great pleasure | 1 | 2 | 3 | 4 | 5 |

*Note: The 3 Italic items (7, 9, 16) have lower outer loading values and are deleted from the final model.*

**3- Consumer-Based Brand Equity** (Mishra, Dash, Malhotra, & Cyr, 2015)

Strongly Agree: 1 Agree: 2 Neutral: 3 Disagree: 4 Strongly Disagree: 5

| Sr # | Items | Strongly Agree | Agree | Neutral | Disagree | Strongly Disagree |
| --- | --- | --- | --- | --- | --- | --- |
| 1 | It makes sense to choose the 3D printed phone accessory (casing/holder) of this brand instead of any other, even if they are the same | 1 | 2 | 3 | 4 | 5 |
| 2 | Even if another 3D printed accessory has the same features/options as this brand’s accessory, I would prefer to choose this one. | 1 | 2 | 3 | 4 | 5 |
| 3 | If there is another brand’s 3D printed accessory as good as this one, I still prefer to choose this accessory (casing/holder) | 1 | 2 | 3 | 4 | 5 |
| 4 | If another brand’s 3D printed accessory is not different from this one in any way, it seems smarter to choose this accessory (casing/holder) | 1 | 2 | 3 | 4 | 5 |
